# Epidemiology of medication-related problems in children with kidney disease

**DOI:** 10.1007/s00467-014-2982-5

**Published:** 2014-10-29

**Authors:** Norkasihan Ibrahim, Ian Chi Kei Wong, Stephen Tomlin, Manish D. Sinha, Lesley Rees, Yogini Jani

**Affiliations:** 1 Centre for Paediatric Pharmacy Research, School of Pharmacy, University College London, London, UK; 2 Pharmacy Department, Paediatric Institute, General Hospital Kuala Lumpur, 50586 Jalan Pahang, Kuala Lumpur, Malaysia; 3 Centre for Safe Medication Practice and Research, Department of Pharmacology and Pharmacy, Li Ka Shing Faculty of Medicine, University of Hong Kong, Hong Kong, China; 4 Pharmacy Department, Evelina London Children’s Hospital, Guy’s and St. Thomas’ Hospital NHS Foundation Trust, King’s Health Partners, London, UK; 5 Department of Paediatric Nephrology, Evelina London Children’s Hospital, Guy’s and St. Thomas’ Hospital NHS Foundation Trust, King’s Health Partners, London, UK; 6 Department of Nephrology, Great Ormond Street Hospital NHS Foundation Trust, London, UK; 7 Pharmacy Department, University College London Hospital NHS Foundation Trust, London, UK; 8 Li Ka Shing Faculty of Medicine, University of Hong Kong, 2/F, 21 Sassoon Road, Laboratory Block, Faculty of Medicine Building, Hong Kong, China

**Keywords:** Paediatric nephrology, Medication safety, Prescribing errors, Pharmacotherapy, Drug-related problems

## Abstract

**Background:**

Medication-related problems (MRPs) are the undesirable effects of pharmacotherapy that can potentially lead to harm. The epidemiology of MRPs in paediatric renal patients is unknown. We aimed to characterise MRPs in this population at two tertiary care hospitals in the UK.

**Methods:**

Prescription charts for children (≤18 years) were reviewed to identify MRPs, and characterised using a specific proforma with a standard operational definition. MRP predictors were evaluated by logistic regression and severity was assessed using a validated scale.

**Results:**

Two hundred and sixty-seven MRPs were identified from 266 prescription chart reviews. The incidence was 51.2 % (203 MRPs, 166 charts; 95 % CI 43.2–60.6 %) of hospitalised patients and 32 % (64 MRPs, 100 charts; 95 % CI 22.9–41.1 %) in outpatients. The number of prescribed medications was the only independent predictor during inpatient treatment (OR 1.06, 95 % CI 1.02–1.10,* p* = 0.002) with no significant predictors identified at outpatient clinics. The severity level of the MRPs was minor: 53.9 %, (144 out of 267); or moderate: 46.1 %, (123 out of 267). Sub-optimal drug effect was the predominant MRP (inpatient: 68 %; outpatient: 39 %). Prescribing error and patients' medicine-taking behaviour were the main contributory factors. The majority of the MRPs in the inpatient setting were resolved.

**Conclusion:**

Many factors are associated with MRPs in children; the associations are cumulative and interdependent. Investment in preventive strategies and extending the support from the acute health care setting into the community are invaluable for optimising pharmacotherapy.

## Introduction

Medications are prescribed with the intention of benefiting the patient. However, the use of medications can be undesirable and potentially lead to harm. These undesirable effects are known as medication-related problems (MRPs). A MRP is defined as an event involving pharmacotherapy that interferes with the patient experiencing an optimal outcome of medical care [1]. Medications for patients with kidney disease are specialised and complex. In those with chronic kidney disease (CKD), the relevance of optimal pharmacotherapy management is even more critical, thus putting this group of children at risk of developing MRPs. The National Kidney Foundation/Kidney Dialysis Outcome Quality Indicator (NKF/KDOQI) guideline recommends that a medication review should be performed for children and adolescents at all visits for the prevention of MRPs such as inappropriate doses, inadequate therapeutic monitoring and potentially adverse drug effects on the kidney or disease complications [2].

Medication-related problems are common in children: recent studies have reported that the incidence in the UK is 39.4 % in hospitalised children [3] and 2.7 % of children on medications were admitted to the emergency departments in the UK [4]. However, these studies did not include paediatric renal patients. Current management of MRPs in this group is based on what has been learned from adult studies [5], which may be unrepresentative of the paediatric population. We have previously reported on the paucity of data on the epidemiology of MRPs in children with kidney disease and suggested further studies for proactive strategies in medication safety [5]. This current study aimed to determine the characteristics of MRPs and potential risk factors for their occurrence in children with kidney disease at the tertiary renal centres.

## Materials and methods

### Setting and study subjects

Two observational cohort studies were conducted at the renal unit of the Evelina London Children’s Hospital (ELCH) and Great Ormond Street Hospital for Children (GOSH). Study 1 was conducted in an inpatient setting at ELCH and GOSH and study 2 was conducted in the renal outpatient clinics at ELCH. Approval was obtained from the Research Ethics Committee, UK. Both hospitals are two of the largest children’s renal centres and provide tertiary care to approximately 40 % of children requiring renal replacement therapy (RRT) in the UK [6]. The renal units are led by teams of paediatric nephrologists, supported by a team of healthcare professionals including specialist renal nurses, renal pharmacists and nutritionists. The cases seen in both units include all aspects of paediatric renal disease, including RRT.

#### Study 1, inpatient setting

The clinical team in both hospitals conducted daily a full patient review and clinical rounds. Clinical pharmacy practice is established as standard care in the inpatient setting; this enabled the pharmacists to review prescription charts as a standard clinical routine. Prescription chart review was conducted using paper documentation in ELCH and from an electronic prescribing system (ePS) in GOSH.

#### Study 2, outpatient clinic setting

The renal outpatient clinics in ELCH operate daily on weekdays. Patients’ clinic notes and medication lists were documented on a multidisciplinary electronic medical record (eMR). Unlike the inpatient setting, clinical pharmacy practice is not part of the standard care and thus, prescription chart review was not routine for all patients attending the clinic.

The inclusion criteria for both studies were the same: all children aged 18 years and younger, who had received at least one long-term (non-acute) medication. Additionally, children who were included in the inpatient study had to have been hospitalised for at least 24 h.

### Data collection

#### Study 1, inpatient study

Data were collected for hospitalised patients at ELCH and GOSH from 1 December 2011 to 1 September 2012. At each site, one pharmacist was responsible for the detection of MRPs using a structured tool and prospective prescription chart review method during routine clinical practice. Recommendations on the resolution of the MRPs were discussed with the clinical team during clinical rounds; this method has been used in previous paediatric MRP studies [3, 4, 7, 8].

#### Study 2, outpatient clinic study

Data were collected at ELCH from 18 February to 18 September 2013. As clinical pharmacy practice was not a standard care at the outpatient clinic and in order to ensure the consistency of data, one researcher was responsible for identifying MRPs from the eMR, using a structured approach. In the event that an MRP with potential harm to the patient was identified, the researcher would alert the clinical team.

The two pharmacists who were involved in collecting the data in the inpatient setting had received similar professional training and each had more than 10 years’ experience in tertiary paediatric renal pharmacy practice. The researcher is also a qualified pharmacist trained in renal pharmacy, but not working in the capacity of a pharmacist within the Trusts. The pharmacists and the researcher received training on the definition and characteristics of MRPs before initiation of the research. All MRPs were recorded onto a proforma using a standard code and operational definition (Appendix 1). All prescribed medications associated with MRPs were classified according to the World Health Organisation Anatomical Therapeutic Chemical (WHO-ATC) system.

All different medications prescribed throughout the patients’ hospital stay (in study 1) and all medications listed in the patient’s clinic notes (in study 2) were recorded.

### Incidence and risk factors

The incidence of MRPs in the study cohort was defined as the number of patients with at least one MRP identified during prescription chart review, divided by the total number of patients reviewed multiplied by 100. For MRP incidence and risk factor calculations, only the first event of MRP identified during review was considered for investigating the association between MRP incidence and potential risk factors.

### Analysis of MRP severity

Similar to previously published paediatric MRP studies [3, 9] this project adopted a validated severity scoring tool for medication errors [10]. The MRPs were independently assessed by four health care professionals comprising a paediatric nephrology consultant, paediatric consultant pharmacist, medication safety pharmacist and a specialist renal nurse. The MRPs were assessed in terms of clinical significance, with scores ranging from 0 to 10 using a visual analogue scale, where 0 represented a case with no potential harm and 10 represented a case that would result in death. An MRP was considered minor (unlikely to have any adverse effects) if the score was < 3, moderate (likely to cause some adverse effects or interfere with therapeutic goals) if the score was between 3 and 7, or severe (likely to cause lasting impairment or death) if the score was > 7. The mean severity score (μScore) from all assessors was the final score assigned to each MRP.

### Classification of medications and MRPs

The medications that were associated with MRPs were classified according to the WHO-ATC system. The identified MRPs were documented in a specified proforma adapted from the Pharmaceutical Care Network Europe (PCNE) MRP Classification Scheme [11]. The operational definition of the types and contributory factors of MRPs is available in Appendix 1.

### MRP resolution

Prescription review was not standard clinical practice in the outpatient clinic; thus, corrective measures and resolution rates could only feasibly be evaluated for MRPs identified during study 1, in the inpatient setting. A MRP was defined as “solved” on occasions when actions were taken before causing any harm to patients or actions were taken to solve an ongoing MRP.

### Statistics

Data were analysed using the Statistical Package for Social Science (SPSS) version 21 and presented as percentages (%), mean with standard deviation (SD) or median with interquartile range (IQR 1–3) and odds ratios (OR) with 95 % confidence interval (CI). For the descriptive analysis of patient and MRP characteristics, Chi-squared (*χ* ^2^), Kruskal–Wallis, Rank and Mann–Whitney tests were used as appropriate. In all statistical tests* p* values of less than 0.05 were considered statistically significant.

Multivariate logistic regression was performed to assess the impact of predictors on the likelihood MRPs would occur among the study population. The regression worked around one binary outcome (at least one MRP identified: yes/no) and six independent variables: age (years), gender, number of medications prescribed (Rx) and types of RRT (dialysis, post-transplant and non-RRT). The variable Rx refers to the number of different medications that were prescribed during the data collection period. Additional independent variables analysed in the inpatient study were length of hospital stay in days (LOS) and types of ward admission (elective or non-elective). Elective admission refers to scheduled admission to the ward for inpatient treatment or procedure. Non-elective admission refers to cases transferred from other wards or hospitals for continuation of care. The factors that were analysed in the regression analyses included those found to be relevant in the literature and of pathophysiological significance [3, 12–14].

## Results

A total of 227 patients fulfilled the inclusion criteria and 267 MRPs were identified (*Inpatient,* n = 127 patients, 203 MRPs (mean 1.2 MRPs per patient (SD 2); *Outpatient,* n = 100 patients, 64 MRPs (mean 0.6 MRPs per patient (SD 1.4)). All MRPs were included for analyses. The incidence of MRPs in the inpatient cohort was 19.2 % higher than the Outpatient cohort (51.2 %, 95 % CI 43.2 – 60.6 % vs. 32 %, 95 % CI 22.9 – 41.1 %, p = 0.04). The study results are summarised in Table 1.

**Table 1:** Summary of results for study 1 (inpatient setting) and study 2 (outpatient renal clinic)

	Study 1: inpatient setting	Study 2: outpatient renal clinic
Number of patients recruited	127	100
Gender (male)	53.5 %,* n* = 68	55 %,* n* = 55
Median age (IQR) of children with MRPs	5.0 (1.3–11.9)	10.3 (5.2–13.8)
MRP incidence per patient reviewed (95 % CI)	51.2 % (43.2–60.6)	32 % (22.9–41.1)
Significant risk factor for the occurrence of MRPs	The numbers of medicine prescribed per child	None identified
Total MRPs identified (*n* = 227)	203 MRPs	64 MRPs
MRP severity level	Minor 68 %, 138 out of 203	Minor 9.4 %, 6 out of 64
	Moderate 32 %, 65 out of 203	Moderate 90.6 %, 58 out of 64
Predominant MRPs	Sub-optimal drug effect 21.7 %, 44 out of 203	Sub-optimal drug effect 39.1 %, 25 out of 64
MRP resolution rate	Solved DRPs 96 %, 195 out of 203	Not evaluated

*MRP* medication-related problem,* DRP* drug-related problem

### Characteristics of the study population

The majority of patients were male. Inpatients were younger than the outpatient cohort (inpatient: median 6.6 years [IQR 1.8–12.5]; outpatient: median 9.7 (IQR 5.7–14.2),* p* = <0.001). Inpatients who developed MRPs, compared with those who did not, had a longer length of hospital stay (median 9 vs 4 days,* p* < 0.001), were on dialysis or had kidney transplant (*p* = 0.001) and more medications prescribed (*p* < 0.001; Table 2). Of the 127 inpatients, 6 were diagnosed with acute kidney injury (AKI). Of the 6 AKI cases, 1 was secondary to sepsis and another was due to the use of radio-contrast agent. The remaining 4 cases could not be confirmed to have any association with the use of medications as they had unknown causes.

**Table 2:** Patient demographic characteristics

	Inpatients (*N* = 127)			Outpatient clinic (*N* = 100)		
	MRPs^b^	No MRPs	*p* value	MRPs^b^	No MRPs	*p* value
Gender			0.44			0.76
Male	37 (29.1)	31 (24.4)		17 (17.0)	38 (45.0)	
Female	28 (22.0)	31 (24.4)		15 (15.0)	30 (37.0)	
Median age	5.0 (1.3–11.9)	8.1 (2.1–13.2)	0.22	10.3 (5.2–13.8)	9.3 (5.7–14.4)	0.85
Median length of hospital stay (days)	9 (4–20)	4 (3–7)	<0.001	NA	NA	NA
Type of ward admission			0.45	NA	NA	NA
Elective	41 (32.3)	35 (27.6)				
Non-elective	24 (18.9)	27 (21.3)				
Renal replacement therapy			0.001			0.38
Dialysis	20 (15.7)	8 (6.3)		13 (13.0)	22 (22.0)	
Kidney transplant	18 (14.2)	11 (8.7)		7 (7.0)	12 (12.0)	
No RRT	27 (21.3)	43 (33.9)		12 (12.0)	34 (34.0)	
Median number of medicines prescribed per child^a^	28 (13–15)	10 (7–19)	<0.001	5 (3–7)	5 (3–7)	0.55

Data are median (IQR 1–3) or frequency (% of* N*)

*RRT* renal replacement therapy, *MRP* medication-related problem

^a^Median number of medicines prescribed throughout hospital stay

^b^At least one MRP identified during prescription review

### Risk factors for MRPs

The number of medications prescribed per child was the single significant predictor for MRPs at the inpatient setting (OR 1.06, 95 % CI 1.02–1.10,* p* = 0.002). The odds ratio of 1.06 indicates that the chance of having an MRP is 6 % higher with every additional medication. There was a trend toward a higher prediction of MRPs in patients on dialysis compared with those not on dialysis, but this finding was not statistically significant. None of the predictors analysed here was statistically significant for the outpatient cohort.

### MRP severity assessment

The 64 MRPs identified from the outpatient clinic scored higher on the severity scale compared with the 203 MRPs identified in the inpatient setting (μScore range: outpatient clinic: 2.1–5.8; inpatient 0.1–6.8).

### Medications associated with MRPs

The groups of medicines most often associated with MRPs were those used for “alimentary tract and metabolism”, “systemic anti-infective” and “blood and blood forming organs” (Table 3). MRPs in inpatients were commonly reported with the use of nystatin 7.9 %, 16 out of 203), paracetamol (7.4 %, 15 out of 203) and ranitidine (5.4 %, 11 out of 203), whereas MRPs in outpatients were associated with prednisolone (15.6 %, 10 out of 64), sodium bicarbonate (9.4 %, 6 out of 64) and alfacalcidol (7.8 %, 5 out of 64).

**Table 3:** Medications associated with MRPs

WHO ATC code	Study 1: inpatient *n* (% of 203)	Study 2: outpatient clinics *n* (% of 64)
(A) Alimentary tract and metabolism^a^		
Nystatin, ranitidine, alfacalcidol, calcium carbonate^b^	52 (25.6)	12 (18.8)
(B) Blood and blood-forming organs^a^		
Acetylsalicylic acid, erythropoietin, electrolytes (e.g., sodium bicarbonate/chloride)^b^	27 (13.3)	15 (23.4)
(C) Cardiovascular system		
Enalapril, furosemide, nifedipine^b^	10 (4.9)	2 (3.1)
(D) Dermatological		
Fusidic acid, gentamicin, mupirocin^b^	4 (2)	0
(G) Genitourinary system and sex hormones		
Oxybutinin^b^	2 (1)	0
(H) Systemic hormonal preparations, excluding sex hormones		
Steroids-based products (e.g., prednisolone, methylprednisolone), octreotide, levothyroxine^b^	15 (7.4)	10 (15.6)
(J) Anti-infectives for systemic use^a^		
Penicillins, cephalosporins, vaccines^b^	49 (24.1)	11 (17.2)
(L) Antineoplastic and immunomodulating agents		
Tacrolimus, mycophenolic acid, ciclosporine, cyclophosphamide^b^	11 (5.4)	10 (15.6)
(M) Musculo-skeletal system		
Pamidronic acid^b^	1 (0.5)	0
(N) Nervous system		
Paracetamol, morphine, codeine	23 (11.3)	3 (4.7)
(P) Antiparasitic products, insecticides and repellents		
Levimasole^b^	1 (0.5)	1 (1.6)
(R) Respiratory system		
Cyclizine, fluticasone, cetrizine^b^	7 (3.4)	0
(S) Sensory organs		
Dexamethasone and anti-infectives^b^	1 (0.5)	0
(V) Various		
Polystyrene sulfonate^b^	1 (0.5)	0

*WHO ATC* World Health Organisation Anatomical Therapeutic Chemical system

^a^The predominant groups

^b^The most common medicines reported in each group

### The types and contributory factors of MRPs

Sub-optimal drug effect was the predominant MRP identified in both clinical settings; however, their contributory factors were distinct. The characteristics of the MRPs are presented in Table 4.

**Table 4:** Types of medication-related problems (MRPs) identified in the study cohort by the main and sub-categories^a^

MRP characteristics		Study 1, inpatient setting		Study 2, outpatient clinic	
		*n* (%)		*n* (%)	
Types of MRPs	P1.1 No effect of drug treatment	3/203	(1.5)	0/64	
	P1.2 Sub-optimal effect	44/203	(21.7)	25/64	(39.1)
	P1.4 Untreated indication	20/203	(9.9)	1/64	(1.6)
	P2.1 Non-allergic reaction	22/203	(10.8)	11/64	(17.2)
	P2.2 Allergic drug reaction	3/203	(1.5)	1/64	(1.6)
	P2.3 Toxic adverse reaction	39/203	(19.2)	2/64	(3.1)
	P3.1 Unnecessary treatment	41/203	(20.2)	0/64	
	P4.1 Patient dissatisfaction	1/203	(0.5)	0/64	
	P4.2 Drug administration problems	30/203	(14.8)	19/64	(29.7)
	P4.3 Delay in treatment	0/203		5/64	(7.8)
Contributory factors for MRPs^b^	C1 Inappropriate drug selection	72/399	(18.0)	3/73	(4.1)
	C2 Inappropriate drug form	7/399	(1.8)	0/73	
	C3 Inappropriate drug dosage	97/399	(24.3)	4/73	(5.5)
	C4 Inappropriate treatment duration	15/399	(3.8)	0/73	
	C5 Medication errors	181/399	(45.4)	1/73	(1.4)
	C6 Drug supply problems	0/399		8/73	(11.0)
	C7 Patient factors	1/399	(0.3)	31/73	(42.5)
	C8 Other factors	26/399	(6.5)	26/73	(35.6)

^a^The operational definition for the types and contributory factors of MRPs is available in Appendix 1

^b^Full description of the contributory factors for MRPs is available in Appendix 2.

#### Inpatient setting

The predominant MRPs were “sub-optimal drug effect” (21.7 %, 44 out of 203), “unnecessary treatment” (20.2 %, 41 out of 203) and “toxic adverse reaction” (19.2 %, 39 out of 203). The majority of the MRPs were identified and resolved before they caused any harm (55.1 %, 112 out of 203). Prescribing errors in the selection of medications and doses were the main contributory factors for the occurrence of MRPs. The following are examples of cases:

1. Valganciclovir 400 mg once a day was prescribed post-transplant; the dose should have been optimised to 500 mg according to renal function (μScore 2.8).
2. 50 mcg stat dose of intravenous prazosin was prescribed to a neonate, but 500 mcg was administered (μScore 5.5).
3. Intravenous gentamicin for septicaemia was prescribed for a patient with acute renal dysfunction; daily doses of gentamicin were continued for 2 weeks without the serum drug level being monitored (μScore 7.4).

#### Outpatient clinic

The predominant MRPs were “sub-optimal drug effect” (39.1 %, 25 out of 64), “drug administration problems” (29.7 %, 19 out of 64) and “non-allergic adverse drug events” (17.2 %, 11 out of 64). The contributory factors for MRPs were associated with patients’ medicine-taking behaviour and other factors (e.g. unwanted side effects and dependency on feeding tubes for the administration of medications). The following are examples of cases:

1. A patient with vesicoureteric reflux was dependent on the enteral feeding tube. The family struggled to administer iron supplement (and other oral medications) as prescribed (μScore 2.1).
2. Activated vitamin D for the prevention of hyperparathyroidism was prescribed to a patient with advanced kidney failure. The family had problems in obtaining a continuous supply in the community setting (μScore 3.9).
3. A patient had on-going proteinuria, but had been non-compliant with treatment for the past 6 months (μScore 5.8).

### MRP resolution

Of the 203 MRPs identified from the inpatient setting, 96 % (195 out of 203) were resolved as a result of multidisciplinary care. Most MRPs were resolved by changes to the medication selection, doses and dosing frequency. The pharmacists played an important role in providing consultations on medication regimens and 99.5 % of the recommendations (227 out of 228) were accepted by the clinical team.

## Discussion

To our knowledge this is the first study investigating MRPs systematically in children with kidney disease in the UK. We observed that whilst not all predictors for MRPs were significant, our results are particularly important, not only in understanding the characteristics of MRPs in this population, but also in enabling the development of preventive strategies in clinical practice.

It is worth discussing further the differences in the demographic characteristics between study subjects in the inpatient and outpatient setting in the current research. Even though all subjects were children with kidney disease, the majority of those seen at the outpatient clinics were in the pre-dialysis stage. Our study shows that those receiving inpatient treatments require three times the numbers of medications than the outpatient cohort (median 17 vs 5 medications per child). Children receiving inpatient treatment were by definition more ill and this was likely due to more advanced disease or complications of RRT. Many studies in adults with CKD have previously demonstrated that patients at the late stage of CKD and on RRT require more complex pharmacotherapy and are exposed to increased chances of MRPs [15, 16]. There was an association between the occurrence of MRP and the length of hospital stay; however, further research is required to assess causality.

Interestingly, despite having more serious clinical conditions in hospitalised patients, the majority of MRPs were scored as minor (68 %, 138 out of 203) compared with the MRPs identified at the outpatient clinic in which 90.6 % (58 out of 64) were scored as moderate. The possible reason for this lies in the difference of care between tertiary care centres and the community. Problems in the use of medications occurring in inpatients are “active errors” [17]. Similar to previous paediatric medication error research [7, 8, 18, 19] the majority of the “active errors” in the current study were caused by prescribing errors. These errors could rapidly be rectified by the healthcare professionals and were less likely to cause harm as a result of the interventions. As an example, sub-optimal tacrolimus dose in the management of post-kidney transplantation on the ward could be adjusted from post-12-h tacrolimus serum drug levels. Changes to drug regimens are directly monitored and patients receiving inpatient treatment are likely to adhere to the prescribed therapy.

In contrast, drug problems occurring in the community are “latent errors”, most of which are caused by exogenous factors that are beyond the control of healthcare professionals [20]. An example of exogenous factors in the current research was difficulties in obtaining unlicensed and off-label medications from the community.

The types of medications associated with MRPs in our study portrayed the common prescribing pattern in the paediatric population [3, 21, 22]. However, medicines of the “blood and blood forming agents” are unique to MRPs in renal patients [12, 13, 23, 24]. The medications that were more often associated with MRPs were also those more often prescribed. It is also important to note that there are limited data available for the safe and effective doses of most medications used in children [3, 25]. This may contributed to the observed tendency for higher numbers of adverse drug events and other MRPs in this cohort.

It is a challenging task to determine an optimal and safe dose for children with kidney disease owing to their physiology and the altered pharmacokinetic properties of medications [21, 26]. The management of MRPs should be a shared responsibility of all healthcare providers. The MRP classifications and the MRP screening tool used in the present research could also be integrated into the physician’s practice.

Other strategies to reduce the occurrence of MRPs are to integrate medication reconciliation into patients’ medicine management program. This program was also recently highlighted for the care of adult renal patients [27, 28]. In the UK, a survey among paediatric pharmacists found that only 34 % had full medication reconciliation in place [29]. A policy on medication reconciliation on hospital admission and at discharge, including routine outpatient clinic appointments, minimises discrepancies in the transfer of information [3].

In the inpatient setting, we found that most MRPs are largely attributed to prescribing errors. Prescribing errors have been reported to be preventable; thus, having continuous awareness programmes on medication safety in paediatrics remains essential in practice [8, 29–32].

This study reported the rate of MRP occurrence in children who attended the renal outpatient clinic as less than one MRP per patient. Nevertheless, the potential harm as a consequence of these MRPs should not be underestimated. The latent effect of MRPs in this patient group has not been studied. We identified cases of poor treatment outcome due to patient non-adherence. An example of these cases that was highlighted earlier involved a patient with glomerulonephritis leading to persistent proteinuria.

Difficulties in obtaining medication supplies and poor understanding about medications among parents and children are among the factors causing low adherence in CKD [15, 33]. Thus, proper coordination of supplies of medication in the community is also vitally important and should be anticipated when unlicensed and/or off-label medications are prescribed for children. Continuous assessment on changing patients’ cognitive behaviour towards medicines and/or specific clinical outcomes is also important to empower patients’ involvement in managing their medications [15, 32, 34]. As caring for children with kidney disease is a life-long commitment, parents/carers would benefit from a support system to facilitate them in the monitoring, prevention and resolution of MRPs.

The strengths of this research lie in using multiple approaches to identifying MRPs, i.e. a prospective chart review in the inpatient setting and a retrospective chart review in the outpatient clinic. We were aware that the characteristics of MRPs identified in both studies reflect the types of methods used. Thus, appropriate measures had been taken to minimise the variability of data that include the use of a structured proforma with a standard operational definition and training to those involved in collecting the data. This research included two main referral centres for paediatric nephrology in London. Thus, the results may not necessarily be generalisable to other countries with different care settings.

## Conclusion

Medication-related problems in children with kidney disease necessitate a comprehensive approach to their identification and resolution. The MRPs in different clinical settings are unique in their characteristics and levels of severity. Investment in preventive strategies and extending the support from the health care setting into the community are invaluable for optimising pharmacotherapy.

## Appendix 1

**Table 5:** Coding system for the types and contributory factors of medication-related problems (MRPs) and the operational definition of the modified Pharmaceutical Care Network (PCNE) MRP classification version 6.2

Main category: types of MRPs	Codes	Sub-categories	Operational definition
Drug effect	P1		There is a (potential or manifested) problem with the (lack of) effect of the pharmacotherapy
	P1.1	No effect of treatment/therapy failure	There is neither improvement nor worsening of the patient’s symptoms
	P1.2	Sub-optimal drug effect	There is improvement in the patient’s symptoms, but not to the intended target
	P1.3	Wrong effect of drug treatment	NA
	P1.4	Untreated indication	There is a symptom (or an anticipated symptom) requiring drug therapy that is not treated
Adverse drug events	P2		Patient suffers, or will suffer, from an adverse drug event
	P2.1	Non-allergic adverse reaction	An unintended pharmacological effect from an adverse drug event not suspected as an allergic reaction (or toxic effect) commonly known to be related to the prescribed drug at doses normally used for the intended indication (e.g. side effects, intolerable intended pharmacological effect, e.g. hypotension from the use of antihypertensive agent)
	P2.2	Allergic drug reaction	An unintended pharmacological effect from an adverse drug event suspected as an allergy reaction or toxicity, commonly known to be related to the prescribed drug at doses normally used for the intended indication (e.g. rash and penicillin)
	P2.3	Toxic adverse reaction	An unintended pharmacological effect related to the drug at doses higher than maximum dose normally used for the intended indication or adverse effect cause by accumulated doses
Treatment costs	P3		The drug treatment is more expensive than necessary
	P3.1	Drug treatment more costly than necessary	There is an alternative drug that is cheaper, but is not being used
	P3.2	Unnecessary drug treatment	The drug that is newly (or previously) prescribed is not required (or no longer required)
Other	P4		Other causes not specified above
	P4.1	Patient dissatisfied with therapy despite optimal clinical and economic treatment outcomes	Self-explanatory
	P4.2	Drug administration problems	Difficulties in administering the appropriate drug at the correct doses to the intended patient (e.g. paracetamol suppository (540 mg) was prescribed, but the preparation available at the dispensary is a 240-mg suppository; incomplete instructions of drug administration; any circumstance that hinders drug administration)
	P4.3	Delay in treatment	Self-explanatory
Drug selection	C1		The cause of the DRP is related to the selection of the drug
	C1.1	Inappropriate drug (including contra-indication)	The wrong drug is selected or the selected drug is contraindicated for the patient.
			Wrong drug is, for example, a patient who is supposed to be on antibiotic A, but is administered antibiotic B
			Contraindicated drug use is, for example, a patient who received a drug to which he had previously experienced an allergy reaction
	C1.2	Inappropriate combination of drugs	The selected drug interacts (or has the potential to interact) with another drug(s), food or device
	C1.3	Inappropriate duplication of therapeutic group or active ingredient	More than one drug of the same therapeutic group or active ingredient is used concurrently
	C1.4	Indication for drug treatment not noticed	The drug that is indicated to treat a symptom is not used because the existence of the symptom is not noticed
	C1.5	Too many drugs prescribed for an indication	More than the necessary drugs are used for treating the same symptom(s)
	C1.6	More cost-effective drug available	An alternative drug that is cheaper and as effective (or more effective) is not used
	C1.7	Synergistic/preventive drug required and not given	A drug that is required to enhance the existing treatment (synergistic effect) or to prevent the development of another symptom is not used
	C1.8	New indication for drug treatment presented	The drug has a new indication that requires a change of dosing regimen (e.g. steroid maintenance dose in post-transplantation and pulse doses in acute rejection)
Drug form	C2		Inappropriate drug form
	C2.1	Inappropriate drug form	Inappropriate drug form and/or formulation
Dose selection	C3		The cause of the DRP is related to the selection of the dosage schedule
	C3.1	Drug dose too low	Dose is insufficient to achieve the therapeutic outcome
	C3.2	Drug dose too high	Dose is more than necessary to achieve the therapeutic outcome
	C3.3	Dosage regimen not frequent enough	Dosing frequency is insufficient to achieve the therapeutic outcome
	C3.4	Dosage regimen too frequent	Dosing frequency is more than necessary to achieve the therapeutic outcome
	C3.5	No therapeutic drug monitoring	Serum level for a drug with a narrow therapeutic index, not monitored
	C3.6	Pharmacokinetic problem requiring dose adjustment	Changes in renal function requiring dose adjustment
	C3.7	Deterioration/Improvement of disease state requiring dose adjustment	Changes to disease state requiring dose adjustment
	C3.8	Dose difficult to measure	Prescribed dose difficult to measure
Treatment duration	C4		The cause of the DRP is related to the duration of therapy
	C4.1	Treatment duration too short	Treatment duration is shorter than necessary
	C4.2	Treatment duration too long	Treatment duration is longer than necessary
Medication errors	C5		Mishaps or accidents during at any stage of drug handling, prescribing, transcribing, dispensing and administering
	C5.1	Inappropriate timing of administration and/or dosing intervals	Error in the process of drug administration
	C5.2	Drug underused/under-administered	Error in the process of drug administration
	C5.3	Drug overused/over-administered	Error in the process of drug administration
	C5.4	Drug not taken/administered at all	Error in the process of drug administration
	C5.5	Wrong drug taken/administered	Error in the process of drug administration
	C5.6	Drug abused (unregulated overuse)	Self-explanatory
	C5.7	Patient unable to use drug/drug form as directed	Moved to category patient factor (C7.5)
	C5.8a	Prescribing error in decision making	Error in deciding treatment
	C5.8b	Prescribing error in prescription writing	Error in writing prescription
	C5.9	Dispensing error	Error in dispensing the prescribed drug
	C5.10	Dilution error	Error in the process of diluting a drug to its prescribed concentration
Drug supply	C6.1	Prescribed drug not available	Prescribed drug not available for use
	C6.2	Difficulty in obtaining repeat prescription from the community	There is a problem in obtaining repeat prescription(s) from the general practitioner (GP) or the community pharmacy
Patient factor	C7		The cause of the DRP can be related to the personality or behaviour of the patient
	C7.1	Patient forgot to use/take drug	Self-explanatory
	C7.2	Patient used unnecessary drug	Self-explanatory
	C7.3	Patient took food that interacts with the prescribed drug(s)	Self-explanatory
	C7.4	Patient stored drug inappropriately	Self-explanatory
	C7.5	Patient refused to take the drug	Self-explanatory
	C7.6	Patient unable to use the drug	Self-explanatory
	C7.7	Patient (parent/carer) forgot to obtain repeat prescription(s) from the community	Self-explanatory
	C7.8	Poor understanding of treatment plan and medications	Self-explanatory
Others	C8		Other causes not specified above
	C8.1	Poor medication reconciliation	Discrepancies between the patient’s own drugs with those prescribed on admission.
			Discrepancies between drugs planned to take home and the ones on discharge prescriptions
	C8.2	Unwanted side effects	Known undesirable effect of a drug other than the intended therapeutic effects
	C8.3	Inappropriate drug administration site/route	Wrong site and/or route for the prescribed drug
	C8.4	Dependent on enteral feed tubes	The patient is dependent on enteral feeding tubes for the administration of medicines
	C8.5	Difficulty in obtaining information from the general practitioner	Self-explanatory
	C8.6	New dose not altered by the general practitioner	Self-explanatory

*NA* not applicable

## Appendix 2

**Table 6:** Contributory factors for medication-related problems (MRPs) at the inpatient and outpatient renal clinic*

	Inpatient (*N*_1_ = 399)		Outpatient (*N*_2_ = 73)	
Contributory factors for MRPs	*n* (%)		*n* (%)	
C1 Drug selection	72	(18.0)	3	(4.1)
C1.7 Synergistic/preventive drug not prescribed	20	(5.0)	1	(1.4)
C1.9 No indication	13	(3.3)	–	--
C1.1 Inappropriate drug	10	(2.5)	1	(1.4)
C1.3 Inappropriate drug duplication	9	(2.3)	1	(1.4)
C1.4 Indication for drug not noticed	7	(1.8)	–	--
C1.5 Too many drugs unnecessarily for the same indication	6	(1.5)	–	--
C1.2 Inappropriate drug combination	5	(1.3)	–	--
C1.6 More cost-effective alternative available	1	(0.3)	–	--
C1.8 New indication	1	(0.3)	–	--
C2 Inappropriate drug form	7	(1.8)	–	--
C3 Drug dosage	97	(24.3)	4	(5.5)
C3.2 Dose too high	23	(5.8)	1	(1.4)
C3.1 Dose too low	17	(4.3)	2	(2.7)
C3.4 Dosage regimen too frequent	15	(3.8)	–	--
C3.7 Deterioration/improvement of disease state	10	(2.5)	–	--
C3.3 Dosage regimen not frequent enough	8	(2.0)	–	--
C3.6 Pharmacokinetic problem requiring dosage adjustment	8	(2.0)	–	--
C3.5 No therapeutic monitoring	3	(0.8)	–	--
C3.8 Dose difficult to measure	13	(3.3)	–	--
C4 Treatment duration	15	(3.8)	–	--
C4.2 Too long	13	(3.3)	–	--
C4.1 Too short	2	(0.5)	–	--
C5 Medication errors	181	(45.4)	1	(1.4)
C5.8a Prescribing error in decision making	104	(26.1)	1	(1.4)
C5.8b Prescribing error in prescription writing	72	(18.0)	–	--
C5.2 Drug over-administered	2	(0.5)	–	--
C5.1 Inappropriate timing of drug administration/dosing intervals	2	(0.5)	–	--
C5.9 Dispensing error	1	(0.3)	–	--
C6 Drug supply	**–**	**–**	8	(11.0)
C6.2 Problems with the process for obtaining repeat prescriptions from the community	-–	–	8	(11.0)
C7 Patient factors	1	(0.3)	31	(42.5)
C7.5 Refused to take medicines	1	(0.3)	6	(8.2)
C7.1 Forgot to take the drug	–	–	10	(13.7)
C7.7 Forgot to ask for refill prescription from community	–	–	1	(1.4)
C7.8 Poor understanding of treatment plan and medications			14	(19.2)
C8 Other factors	26	(6.5)	26	(35.6)
C8.2 Unwanted side effects	14	(3.5)	12	(16.4)
C8.1 Poor medication reconciliation	12	(3.0)	–	--
C8.4 Dependent on NG/PEG for medications	–	–	8	(11.0)
C8.5 Difficult to obtain information from GP	–	–	5	(6.8)
C8.6 New dose not altered by the GP	–	–	1	(1.4)

*NG* nasogastric,* PEG* percutaneous endoscopic gastrostomy
